# Efficacy of Stereotactic Radiosurgery in Patients with Multiple Metastases: Importance of Volume Rather Than Number of Lesions

**DOI:** 10.7759/cureus.1966

**Published:** 2017-12-19

**Authors:** Basem A Dahshan, Malcolm D Mattes, Sanjay Bhatia, Mary Susan Palek, Christopher P Cifarelli, Joshua D Hack, John A Vargo

**Affiliations:** 1 Department of Radiation Oncology, Marshall University, Joan C. Edwards School of Medicine; 2 Department of Radiation Oncology, West Virginia University School of Medicine; 3 Department of Neurosurgery, West Virginia University School of Medicine

**Keywords:** stereotactic radiosurgery, srs, multiple metastases, brain metastases, hippocampal avoidance, clinical practice guideline recommendation, quality of life, neurocognitive function, whole brain radiation therapy (wbrt), gamma knife

## Abstract

The role of stereotactic radiosurgery (SRS) in the treatment of multiple brain metastases is controversial. While whole brain radiation therapy (WBRT) has historically been the mainstay of treatment, its value is increasingly being questioned as emerging data supports that SRS alone can provide comparable therapeutic outcomes for limited (one to three) intracranial metastases with fewer adverse effects, including neurocognitive decline. Multiple recent studies have also demonstrated that patients with multiple (> 3) intracranial metastases with a low overall tumor volume have a favorable therapeutic response to SRS, with no significant difference compared to patients with limited metastases. Herein, we present a patient with previously controlled breast cancer who presented with multiple recurrences of intracranial metastases but low total intracranial tumor volume each time. This patient underwent SRS alone for a total of 40 metastatic lesions over three separate procedures with good local control and without any significant cognitive toxicity. The patient eventually opted for enrollment in the NRG-CC001 clinical trial after multiple cranial recurrences. She received conventional WBRT with six months of memantine and developed significant neurocognitive side effects. This case highlights the growing body of literature supporting the role of SRS alone in the management of multiple brain metastases and the importance of maximizing neurocognition as advances in systemic therapies prolong survival in Stage IV cancer.

## Introduction

Brain metastases are a common cause of cancer morbidity and mortality. As advances in systemic therapy increase the average survival time in cancer patients, more patients are at risk of developing brain metastases. Nevertheless, the best approach to the treatment of patients with multiple metastatic lesions of the brain is still a subject of contention. Prior to the development of stereotactic radiosurgery (SRS), whole brain radiation therapy (WBRT) was the mainstay of treatment for patients with brain metastases. However, the use of SRS without the addition of WBRT has become an increasingly common approach to the treatment of limited brain metastases, as it provides comparable overall survival [1]. It also provides better quality-of-life outcomes than SRS with adjunct WBRT with fewer adverse effects of neurocognitive decline and memory loss [2]. As part of the Choosing Wisely campaign, the American Society for Radiation Oncology (ASTRO) has recommended using SRS alone as the standard of care for limited cranial metastases due to the unnecessary morbidity from the routine addition of WBRT [3].

However, the lack of criteria for the use of SRS to treat patients with multiple metastases makes treatment decisions more complicated. Recent data from Yamamoto, et al. has demonstrated that in cases of low intracranial tumor volume (15 mL or less), patients who received SRS alone as a treatment for five to 10 metastases had outcomes comparable to patients treated for limited metastases [4-5]. Here, we present a case of a patient with multiple cranial recurrences but low tumor volume who was treated with SRS alone. Over the course of two years, the patient had multiple cranial recurrences and eventually opted for WBRT. She was enrolled in a clinical trial and was treated with conventional WBRT and concurrent memantine. She experienced cognitive decline, which has reduced her quality of life. This case highlights the growing body of literature supporting the role of SRS alone in the management of multiple brain metastases and the importance of maximizing neurocognition as advances in systemic therapies prolong survival in Stage IV cancer.

## Case presentation

**Figure 1:**
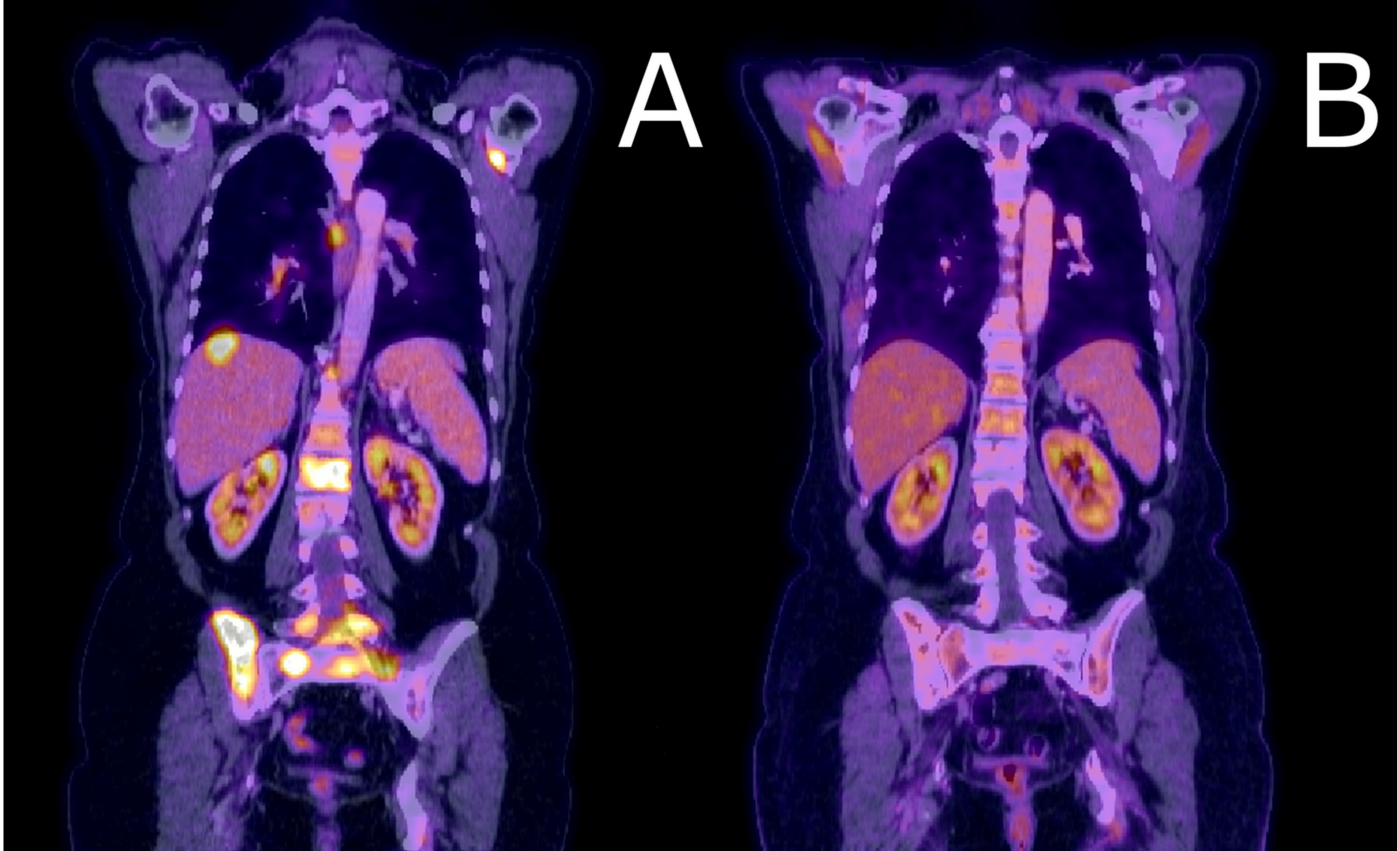
Comparison of PET scans at presentation versus three months after SRS procedure A) At the time of presentation, our patient had extensive extracranial metastases, including the liver, vertebral body, and pelvic bone metastases demonstrated here. The patient underwent systemic treatment and stereotactic radiosurgery (SRS). B) At three months after the initial SRS treatment, the patient demonstrated well-controlled disease. PET: positron emission tomography; SRS: stereotactic radiosurgery

**Figure 2:**
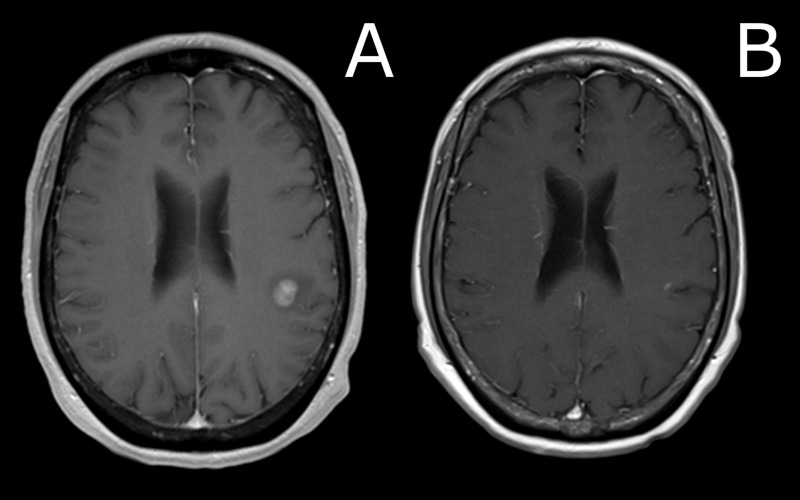
Comparison of brain MRI at presentation versus three months after SRS procedure A) MRI brain imaging revealed two contrast-enhancing lesions in the left frontal lobe (pictured in A); T1 axial image, post-gadolinium contrast) and left cerebellar hemisphere, measuring 1.25 cc and 0.07 cc, respectively. The patient underwent stereotactic radiosurgery (SRS), with 22 Gy delivered to the 50% isodose line to each lesion. B) Three months after SRS, repeat MRI demonstrated resolution of the frontal lobe lesion. MRI: magnetic resonance imaging

**Figure 3:**
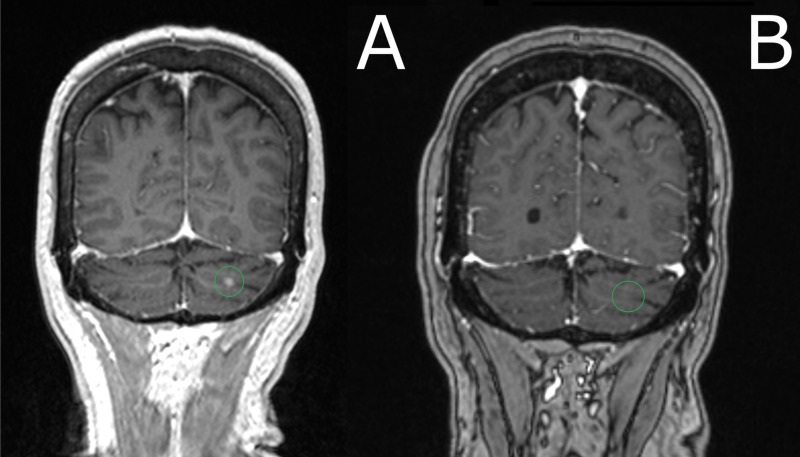
Comparison of brain MRI at presentation (a) versus 3 months after SRS procedure (b) A) MRI brain imaging revealed two contrast-enhancing lesions in the left frontal lobe and left cerebellar hemisphere (pictured here). T1 Axial image, post-gadolinium contrast), measuring 1.25 cc and 0.07 cc, respectively. Patient underwent SRS, with 22 Gy delivered to the 50% isodose line to each lesion. B) Three months after stereotactic radiosurgery (SRS), repeat MRI demonstrated resolution of the cerebellar lesion. MRI: magnetic resonance imaging; SRS: stereotactic radiosurgery

A 57-year-old woman with a previously controlled T1cN0 estrogen receptor-positive/progesterone receptor-positive/human epidermal growth factor receptor-2 (ER+/PR+/HER2-) breast cancer on adjuvant anastrozole presented with fatigue and low back pain. A positron emission tomography/computed tomography (PET/CT) scan was obtained and demonstrated diffuse extracranial metastatic disease (Figure 1). Magnetic resonance imaging (MRI) brain imaging revealed two contrast-enhancing lesions in the left frontal lobe and left cerebellar hemisphere, measuring 1.25 cc and 0.07 cc, respectively (Figures 2-3). On examination, she had no focal neurologic deficits, and her diagnosis-specific Graded Prognostic Assessment score (DS GPA) at diagnosis of brain metastases had an expected median survival of 20.2 months. SRS without WBRT was felt to be appropriate [1, 3]. MRI obtained on the day of the procedure was stable, with a total involved volume of 1.32 cc. The patient underwent SRS with 22 Gy delivered to the 50% isodose line to each lesion and a mean total dose of 0.4 Gy to the whole brain. Around this time, the patient's systemic therapy was changed from anastrozole to single agent protein-bound paclitaxel. Three months after SRS, repeat CT and MRI demonstrated a significant reduction in systemic metastatic burden and cranial lesions (Figures 1-3). However, repeat MRI of the brain six months after the procedure demonstrated seven new punctate metastases. The risks and benefits of SRS and WBRT were reviewed with the patient, but she was very concerned about the risk of neurocognitive side effects and was hesitant to try WBRT. Given her relatively young age, good performance status, favorable response to systemic therapy, and low overall volume of disease, we felt the use of SRS alone over was as effective as WBRT [1, 4], and would reduce the risk of neurocognitive decline [2]. 

On the day of the second procedure, nine months after initial presentation, a high-resolution, double-dose gadolinium-enhanced MRI at time of SRS demonstrated 21 metastases with a total volume of 0.52 cc. Based on the low volume of disease, it was felt that proceeding with SRS alone was still appropriate. All lesions received 20 Gy to the 50% isodose line, except a 0.04 cc brainstem mass which received 16 Gy to the 50% isodose; the mean dose to the whole brain was 0.9 Gy. The patient continued to do well with no symptoms or any focal neurologic deficits noted on follow-up exams. A repeat MRI three months after the second procedure, one year after initial presentation, demonstrated four new sub-centimeter lesions. Treatment options were reviewed with the patient, and SRS was scheduled for the third time. MRI on the day of the procedure demonstrated a total of 17 metastases, with a total volume of 0.83 cc. A dose of 18-20 Gy to the 50% isodose was delivered to all lesions, except a brainstem lesion which received 16 Gy to 50% isodose, with a mean total dose of 0.8 Gy to the whole brain. Repeat scans obtained one month after the third procedure demonstrated no cranial progression or evidence of active malignancy, and the patient had excellent performance status. Shortly after this follow-up scan, the patient was switched to capecitabine due to the development of neuropathy while on the paclitaxel.

Three months after the third SRS procedure, a repeat MRI demonstrated five new foci of enhancement. At this point, the patient opted for WBRT; as with repeated intracranial failure with multiple lesions, it was felt that the benefit of intracranial control might outweigh the risk of neurocognitive side effects. The patient was enrolled in an ongoing Phase III trial (NRG-CC001) involving concurrent and adjuvant memantine added to either conventional or hippocampal-sparing WBRT. She was randomized to the conventional WBRT arm and received 30 Gy in 10 fractions with concurrent memantine. One week after completing WBRT, the patient began complaining of extreme fatigue, a feeling of “brain fog”, feeling very unsteady, a decreased sense of taste, and having little to no appetite. She was started on dexamethasone, but this did not relieve her symptoms. She was managed with supportive care for the next few weeks, and eventually, her fatigue decreased. Two years after initial presentation, the patient is tolerating systemic therapy well. Her symptoms of reduced balance and cognition have improved somewhat in the months since completing WBRT, but they still interfere with her quality of life. She still feels like she is “not herself” and that her overall cognitive abilities are not what they were before WBRT. She also complains of problems with short-term memory and worries that she cannot adequately care for her grandchildren. On the most recent PET and MRI scans (21 months from initial SRS), there are no sites of active malignancy in the body and stable intracranial disease.

## Discussion

Currently, guidelines with regards to the use of SRS for metastases are relatively ambiguous. The most recent ASTRO guidelines on treating brain metastases states that SRS alone is an option for “selected patients” [3]. The guideline cites, among other studies, the EORTC 22952 -26001 trial, which found no overall survival difference between SRS followed by observation versus SRS followed by WBRT; however, only patients with limited (one to three) metastases were eligible [3]. There is a major gap in recommendations regarding the treatment patients with multiple metastases [3]. Despite the apparent evidence gap cited in the guidelines, a growing consensus is emerging for the treatment of multiple metastases with SRS alone. Further evidence has emerged that suggest criteria for SRS alone should consider total tumor burden more so than the absolute number of metastases. The results of the Yamamoto, et al.'s prospective observational study of 1,194 patients found that for patients with ≤ 15 ml total tumor burden, the treatment of five to 10 metastases with SRS alone was non-inferior to the treatment of two to four brain metastases [4]. Yamamoto, et al. further demonstrated in a retrospective study of 720 patients that when patients were matched for clinical factors, such as age, performance status, size of the largest tumor, and control of the primary tumor, the clinical outcomes post-SRS for survival and quality of life were not significantly different for patients with two to nine brain metastases versus patients with 10 or more metastases [5].

Recent data from the Quality of Life after Treatment for Brain Metastases (QUARTZ) trial has also called into question the absolute clinical benefit of WBRT and the role it should play in the treatment of asymptomatic metastases [6]. While it has been hypothesized that new techniques, such as using WBRT with concurrent memantine or hippocampal-sparing WBRT, may help preserve the local control benefit of WBRT [7-8]; these have yet to be substantiated by the results of Phase III clinical trials. The data comparing the addition of memantine to WBRT versus placebo demonstrated borderline statistical significance (P = .059) in long-term preservation of cognitive function as measured by the Hopkins Verbal Learning Test-Revised Delayed Recall [7]. Current data on the relative sparing of neurocognitive function (as a result of hippocampal-sparing techniques) appear promising, although have not yet been confirmed in prospective randomized trials. Gondi, et al. demonstrated a significant reduction of the decline in neurocognitive function at six months post-hippocampal-sparing WBRT versus historical controls [8]. Tsai, et al. have concluded in a prospective study of 40 patients that the rate of decline in neurocognitive function appears to directly correlate with the dose to the hippocampus; specifically, > 40% of the bilateral hippocampus receiving greater than 7.3 Gy was significantly associated with neurocognitive impairment [9].

**Figure 4:**
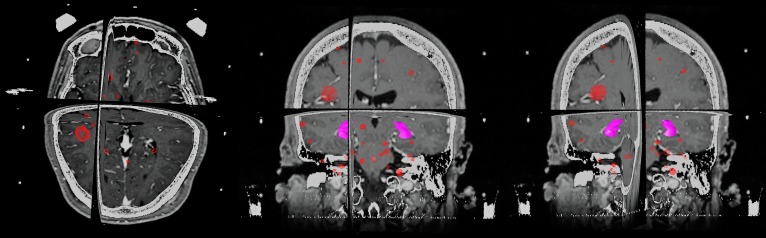
Overview of lesions treated on SRS Pictured here are the combined volumes of three stereotactic radiosurgery (SRS) treatments, with the hippocampus outlined in magenta. Over the course of about 18 months, our patient was treated with SRS for a total of 40 metastases, as described in the case presentation. Post-treatment analysis found a mean total dose of 2.6 Gy received by the hippocampus in three fractions

There are ongoing Phase III trials, such as the NRG-CC001, comparing neurocognitive outcomes versus conventional WBRT. These, as well as a longer follow-up, will be necessary to clearly demonstrate the full extent of this sparing effect and whether it provides comparable local control versus conventional WBRT. In the interim, however, the correlation of declines in neurocognitive function and hippocampal dose begs the question of whether WBRT is the best strategy for hippocampal sparing. Even after treating a total of 40 metastases with SRS, including a right hippocampal lesion, post-treatment analysis of the patient’s SRS plans showed a cumulative mean dose to the hippocampus of 2.6 Gy over three SRS courses (Figure 4). In the prospective study of hippocampal dosimetry by Tsai, et al., the median mean dose to the hippocampus was 5.82 Gy, which demonstrates that SRS may offer superior sparing of the hippocampus compared to “hippocampal-sparing” WBRT even in the setting of multiple metastases [9]. There is also data that this difference is even more pronounced if hippocampi are specifically outlined as a target to avoid in SRS planning [10].

The growing body of evidence of the efficacy of SRS makes it clear that there is a definite role for SRS in patients with multiple metastases. At the same time, it is also apparent that there are still many questions to be answered with regards to the optimal criteria for SRS. Questions remain in terms of if and how the absolute number of metastases affects survival. More importantly, there is still not comprehensive data regarding the upper limit of the total volume of metastases for which SRS is effective, specifically at what point SRS would no longer be an acceptable treatment alternative to WBRT.

## Conclusions

The successful treatment of multiple brain metastases with SRS and the decline in quality of life following the addition of WBRT in this patient highlights that the paradigm of SRS alone being restricted to the treatment < 4 metastases is imperfect. It appears that the total volume of intracranial metastasis is more important than the total number with regards to the efficacy of SRS. It also seems reasonable to question whether patients who are felt to not be SRS candidates receive any significant benefit from palliative WBRT, even with new techniques of delivering the WBRT. As advances in systemic cancer therapy prolong survival, the treatment of brain metastases is an increasingly relevant issue that should not be viewed as a merely palliative measure. Perhaps it is time for the guidelines to be updated in accordance with the emerging data supporting SRS alone for patients with multiple metastases?
